# Minimization of the complications associated with bar removal after the Nuss procedure in adults

**DOI:** 10.1186/s13019-020-01106-1

**Published:** 2020-04-21

**Authors:** Min-Shiau Hsieh, Shao-Syuan Tong, Bo-Chun Wei, Cheng-Chin Chung, Yeung-Leung Cheng

**Affiliations:** 1 Division of Thoracic Surgery, Department of Surgery, Taipei Tzu Chi Hospital, Buddhist Tzu Chi Medical Foundation, No. 289, Jian-Gao RD, Xindian District, New Taipei City, 23143 Taiwan; 2 School of Medicine, Tzu Chi University, Hualien, Taiwan

**Keywords:** Pectus excavatum, Nuss procedure

## Abstract

**Background:**

Pectus bar removal after Nuss repair is associated with the risk of major complications that are underreported. Of these, surgical bleeding is the main concern. Old age and placement of more than one bar are reported risk factors for pectus bar removal. In this study, we presented our experience regarding the modified skills required to minimize complications during bar removal, especially in adult patients.

**Methods:**

Consecutive patients who underwent pectus bar removal as the final stage of Nuss repair between August 2014 and December 2018 were included. The patients were positioned in the supine position. The bar(s) was (were) removed from the left side via the bilateral approach using the previous surgical scars after full dissection of the ends of the bar lateral to the hinge point and after straightening the right end of the bar. Bleeding was carefully checked after removal. An elastic bandage was wrapped around the chest after wound closure to prevent wound hematoma/seroma formation.

**Results:**

A total of 283 patients (260 male and 23 female), with a mean age of 22.8 ± 6.6 years at the time of the Nuss repair were included. The mean duration of pectus bar maintenance interval was 4.3 years (range: 1.9 to 9.8 years). A total of 200 patients (71%) had two bars. The mean estimated blood loss was 11.7 mL (range: 10 mL to 100 mL). Nine patients (3.1%) experienced complications, six had pneumothorax and three had wound hematoma. No major bleeding occurred. Adults and the use of more than one bar were not associated with a significantly higher rate of complications (*P* = 0.400 and *P* = 0.260, respectively).

**Conclusions:**

Adult patients and removal of multiple bars were not risk factors for complications in our cohort. Skill in preventing intraoperative mediastinal traction, carefully controlling bleeding, and reducing the effect of dead space around the wounds could minimize the risk of bleeding complications. A multicentric study or case accumulation is needed to further evaluate the risk factors of removal pectus bar(s).

## Background

Pectus excavatum (PE) is the most common congenital chest wall deformity; it is characterized by a caved-in appearance of the anterior chest [1]. The incidence rate is approximately 0.1% with a male to female ratio of 4:1. It is associated with connective tissue disorders, neuromuscular diseases, and some genetic conditions [1, 2]. In 1998, Nuss and colleagues documented the minimally invasive repair of pectus excavatum (MIRPE), also known as the Nuss procedure, a minimally invasive method for the correction of PE [3]. The reported advantages of MIRPE include the limited number of incisions, short operative time, less blood loss, and shorter duration of hospital stay and recovery. The procedure initially involves the introduction of one or more curved stainless-steel bars behind the sternum to correct the chest wall without resection of the costal cartilages. Considering that the procedure was widely accepted, it has been used extensively in children, adolescents, and adults in the past decade [4–9].

After more than 2 years of correction, the supporting bars need to be removed. In the past, removal of the correction bars was considered to be a simple surgery and patients were even discharged on the day of the removal. However, there have been successive reports of severe complications, including massive hemothorax and death [10–19]. Therefore, some physicians suggested improvements to the surgical equipment and methods used for the removal to reduce surgical complications in a recent decade [11, 17–24]. Although some recent studies have discussed the occurrence of these surgical complications, disparate results were reported in large patient cohorts [11, 18–20, 22]. The statistical data from a large-scale questionnaire survey of physicians from the Chest Wall International Group (CWIG) who have performed such surgeries showed that the incidence of these surgical complications was underestimated and that some severe complications have not been reported [16]. Many previous studies have examined bar removal in teenagers and younger patients after about 2 years of Nuss repair; however, there have been only a few statistical analyses of correction bars inserted in adults after a long time of repair. The CWIG data revealed that more than half of the physicians felt that it was easier to perform surgeries on younger patients. Some modifications of surgical skill for pectus bar removal have been described for reducing the complications [17–24]. Therefore, in this study, we aimed to assess the differences in our modifications on skill and the recent surgical data on pectus bar removal. The methods to decrease intraoperative and postoperative complications were also discussed.

## Methods

### Participants

This retrospective study was approved by the Ethics Committee and the Institutional Review Board (IRB) of the Taipei Tzu-Chi Hospital, Taipei, Taiwan, ROC (IRB No: 08-X-101). The requirement for obtaining patient consent was waived by the IRB because of the retrospective nature of the study. Patients who had PE after the Nuss procedure and underwent pectus bar(s) removal at the Division of Thoracic Surgery of the Taipei Tzu-Chi Hospital in New Taipei City, Taiwan between August 2014 and December 2018 were included. Patient information including age at the time of repair and removal, body mass index (BMI), preoperative chest radiographs, operating time, callus formation around the bars, blood loss, hospital stay duration, and complications were collected from the hospital records.

The medical records of adolescents and adults who underwent surgical removal of the pectus bars after completing the repair for PE and had a maintenance interval of more than 2 years were also collected.

### Surgical techniques for bars removal

**Fig. 1:**
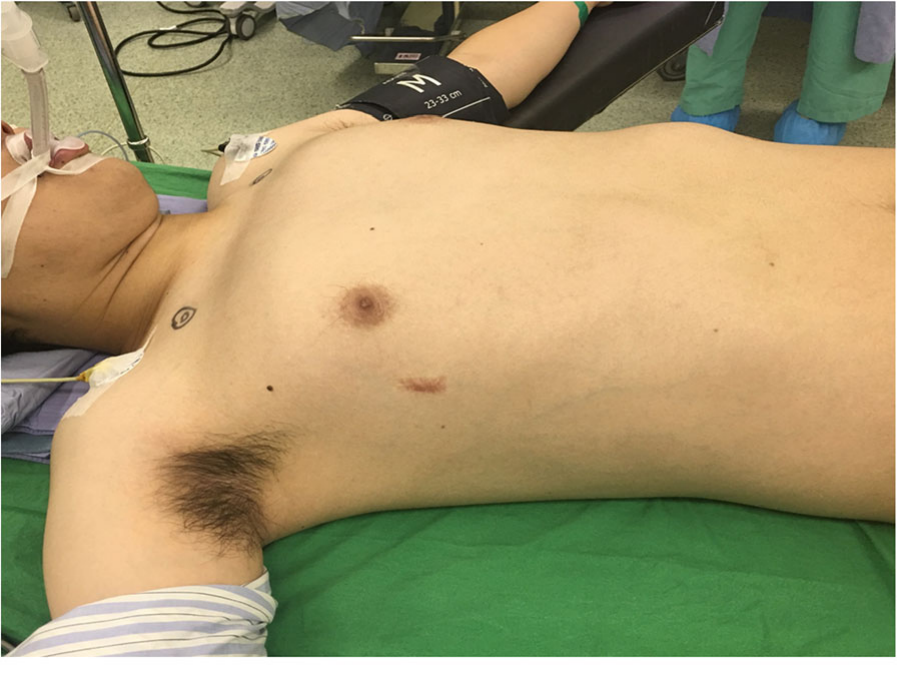
Pectus bar removal. Pectus bar removal was performed with the patient in the supine position and with the upper limbs abducted at about 70° lateral to the trunk

**Fig. 2:**
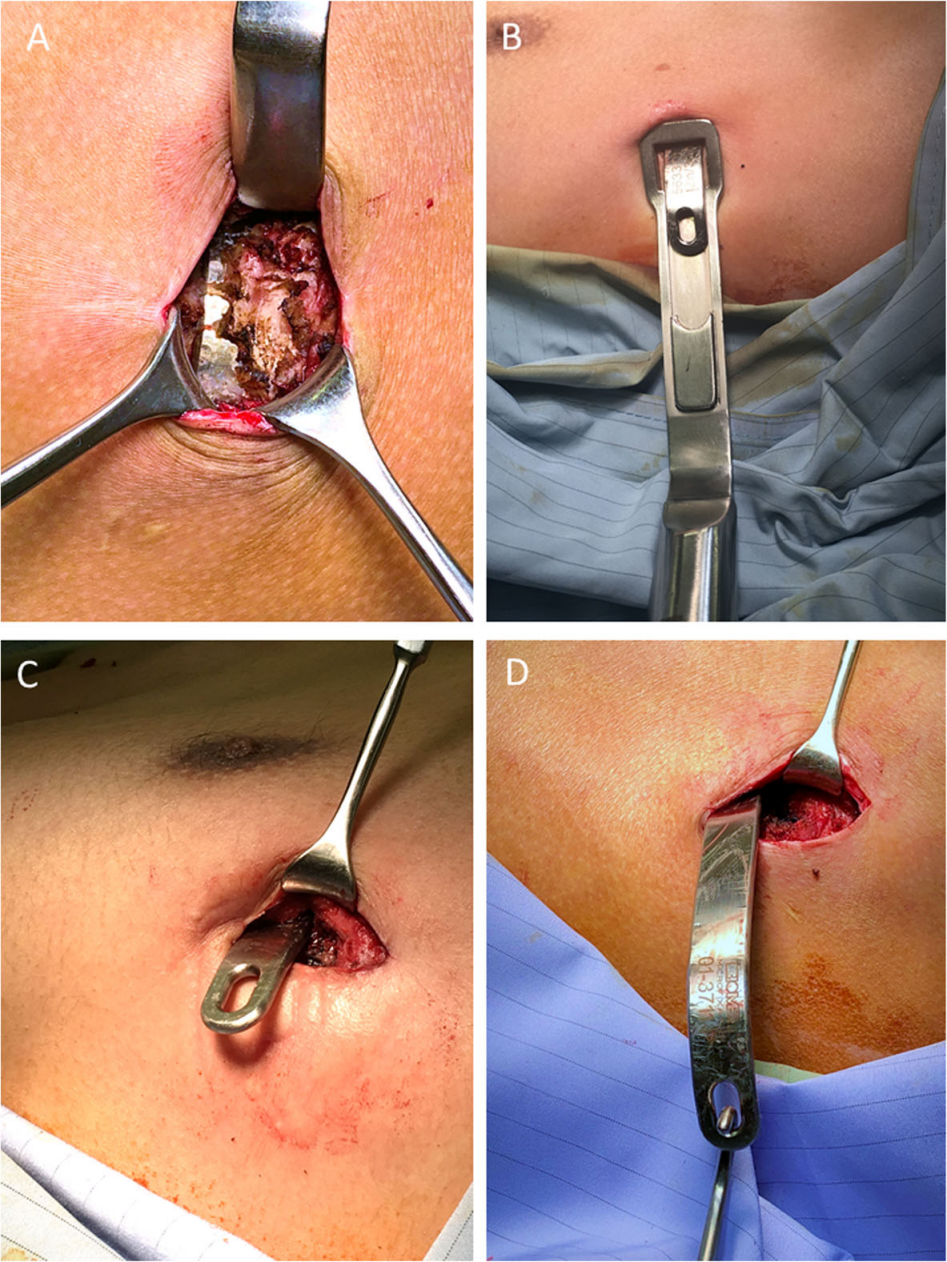
Pectus bar removal in a 25-year-old male patient 4 years after Nuss repair. **a** The right end of the bar incarcerated by a callus (white arrow). **b** The whole right end of the bar external to the hinge point is exposed. **c** The right end of the bar was straightened using the bar bender. **d** Direct removal of the bar from the left end after exposing it along with the anterior curve of the chest wall

**Fig. 3:**
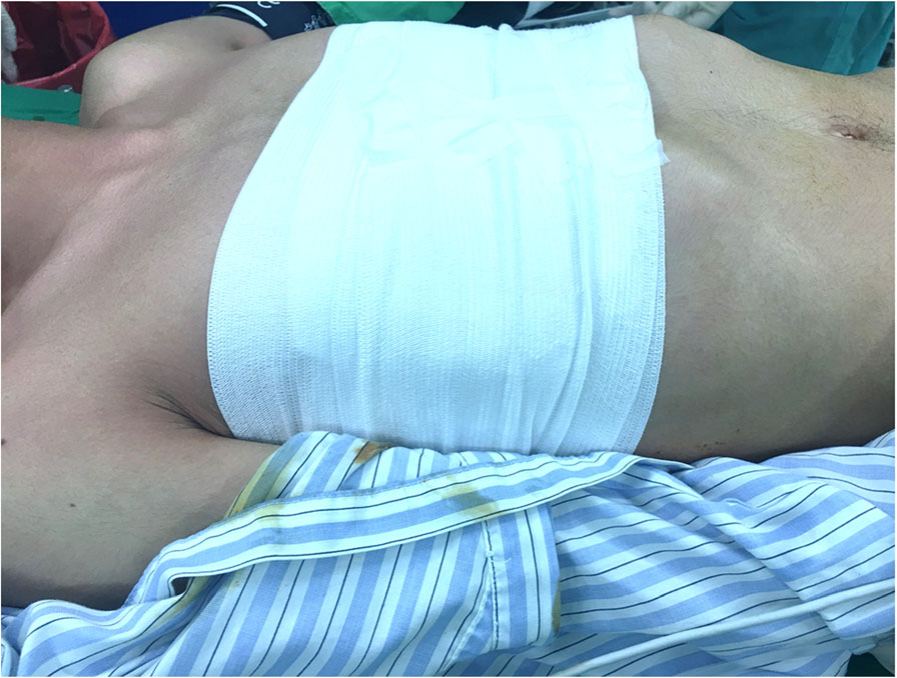
A 6-in. elastic bandage enlacing the thoracic cage. A 6-in. elastic bandage enlacing the thoracic cage was used after the wounds were closed to prevent the formation of hematoma or seroma after bars removal

All patients were placed in the supine position with their arms abducted at about 70° in relation to the body after single-lumen endotracheal tube anesthetic administration (Fig. 1). In general, incisions for the insertion of the bar(s) are made through the old surgical scars. After dissecting subcutaneous tissues, the ends of the bars and the fixation materials were traced and exposed. Subsequently, the fixation materials were removed. Any broken fragments of wire revealed by preoperative chest films were detected and removed using palpable or C-arm fluorography X-rays. If the end(s) of the bar were covered by bony callus (Fig. 2a), the hard callus was cleaned with a rongeur for a total exposure of the ends of the bar lateral to the hinge points (Fig. 2b). After the ends of the bar were exposed, the right end of the bar was partially straightened by the pectus removal bender (Zimmer Biomet, Jacksonville, FL, USA) (Fig. 2c) and the bar was withdrawn through the left side without turning (Fig. 2d). After the bar(s) were removed, bleeding on rough surfaces of the callus was checked and absorbable, hemostatic gauze was inserted to control the local oozing from the callus. The wounds were then closed as usual, in layers and without drainage. After the wounds were covered, a 6-in. elastic bandage was wrapped around the chest to compress the wounds (Fig. 3).

### Postoperative care

A portable chest radiograph was taken at bedside after the operation. Generally, postoperative pain is controlled through intravenous administration of 1–2 doses of nonsteroidal anti-inflammatory drugs. If patients were stable, they were discharged the day after the operation. A chest radiograph would be taken again if a pneumo- or hemothorax occurred. Patients were followed-up 2 weeks postoperatively, then after 6 months, and annually thereafter.

### Statistical analysis

The Kolmogorov-Smirnov test was used to assess the normality of the distribution of the investigated parameters. Only blood loss had a non-normal distribution. The normally continuous data were summarized as mean ± standard deviation (SD) and categorical data were summarized as n (%) by the group. Differences between the two groups were compared using two-sample t-test for continuous data and using Pearson Chi-square test for categorical data. Non-normal continuous data were summarized as mean (range). Differences between the two groups were compared using Mann-Whitney U test. All statistical assessment was two-tailed and was considered significant at *P* < 0.05. Statistical analyses were performed using SPSSⓇ version 22 (SPSS Inc., IMB, USA) software.

## Results

A total of 283 patients who underwent pectus bar removal were included in this study. The male to female ratio was 260:23. Their ages at the initial Nuss repair ranged from 12 to 53, with an average of 22.8 years. In total, 221 patients underwent repair as adults (≥18 years; Group A) while 62 patients underwent repair as adolescents (12–17 years; Group B). The mean pectus bar maintenance interval was 4.3 years (range: 1.9 to 9.8 years) while the mean age upon removal of the bar(s) was 26.9 years (range: 15 to 57 years). A total of 203 patients (71.7%) had two or more bars while 80 patients (28.3%) had only one bar. The mean operative time was 66 min (range: 20 to 187 min) and the mean estimated blood loss was 11.7 mL (range: 10 mL to 100 mL). Ten patients had perioperative blood loss of 50–100 mL. The demographic and perioperative clinical features of Groups A and B are shown in Table 1. Group A had a longer period of correction for Nuss repair than Group B (*P* = 0.010). Overall, the complication rate was 3.2% (9/293), including pneumothorax in six patients (2.1%) and wound seroma/hematoma in three patients (1.1%). The complication rate of Groups A and B showed no significant difference (2.7% vs 4.8%, *P* = 0.400). Others showed no significant association between these two groups. No massive hemothorax, wound hematoma, blood transfusion, or other life-threatening events occurred during or after the operation.

**Table 1:** Clinical analysis of adult (Group A) and adolescent (Group B) patients with pectus excavatum who underwent pectus bar removal after completing repair

	Group A (*n* = 221)	Group B (*n* = 62)	*P* value
Mean age of Nuss repair, year, mean ± SD^1^	25.2 ± 5.1	14.8 ± 2.1	
Mean age of bar(s) removal, year, mean ± SD	29.3 ± 5.4	18.1 ± 3.2	< 0.001
Period of correction, years, mean ± SD	4.4 ± 1.4	3.8 ± 0.7	0.010
Bar number			0.260
One bar, n (%)	59 (28)	21 (40)	
Two or three bars, n (%)	162 (72)	41 (60)	
Operation time, min, mean ± SD	67.9 ± 32.7	59.6 ± 18.4	0.129
Blood loss, mL, mean (range)	12.1 (10–100)	11.6 (10–100)	0.890
Hospital stay, days, mean ± SD	2.9 ± 1	2.6 ± 1	0.610
Complications, n (%)	6 (2.7)	3 (4.8)	0.400
Pneumothorax	4	2	
Hematoma (wound)	2	1	

Normal distribution: mean ± SD; non-normal distribution: mean (range)

Analysis of the association of other surgical factors was demonstrated in Table 2. The results showed that callus formation around the ends of the bars required a significantly longer operative time and caused more perioperative blood loss (*P* = 0.032 and *P* = 0.046, respectively). Patients with a higher BMI (> 22 kg/m^2^) were more likely to have a longer operative time (*P* = 0.048). The other factors showed no significant difference.

**Table 2:**
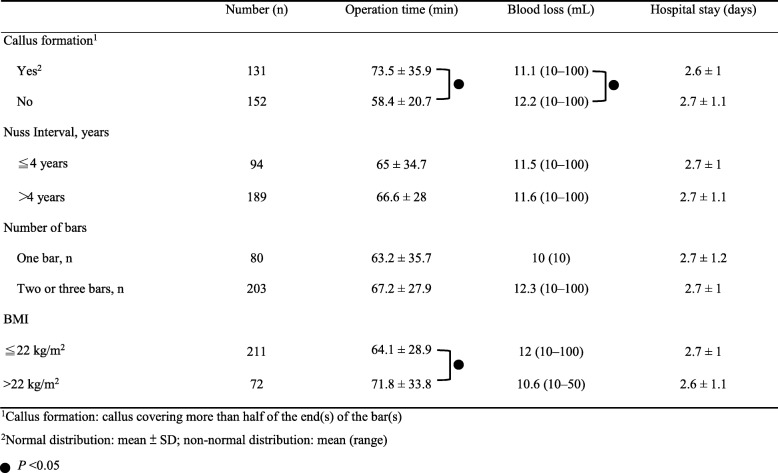
Surgical characteristics of the 283 patients with pectus excavatum who underwent pectus bar(s) removal after the Nuss procedure. ^1^Callus formation: callus covering more than half of the end(s) of the bar(s). ^2^Normal distribution: mean ± SD; non-normal distribution: mean (range). *. *P* < 0.05

## Discussion

Pectus bar removal after the Nuss procedure is considered simple and can be performed as an outpatient procedure. Massive bleeding related to bar removal is rare; however, it may be life-threatening. Myocardial injury, lung laceration, and aortic laceration with massive bleeding after bar removal has been reported [12–15]. Some patients have also died after bar removal; however, this statistic is underreported [16]. Considering patient safety and any unexpected major complications, pectus bar removal is suggested to be performed as an inpatient procedure.

Complications related to bleeding are a major concern during pectus bar removal, although it has only been reported in less than 1% of the cases. Bleeding from the heart, great vessels, chest wall, internal mammary artery, intercostal artery, or callus around the pectus bar has been reported [10, 18]. Patients with a history of intrathoracic infection or pericarditis have higher risks for life-threatening complications [12–14]. Most bleeding could be controlled conservatively; however, internal control of bleeding should be considered if massive intrathoracic bleeding occurred. The other common complication after bar removal is wound seroma/hematoma with an incidence rate of approximately 2.36–11.98% [16, 18, 22]. It can also be from the callus or scar tissues of the wound. Adult patients and patients treated using more than one bar were reported to have a higher complication rate [9, 14]. The major and lethal complications that had been observed with the Nuss Procedure were related to the migration of the bar [3, 17]. The obvious displacement of the pectus bar would increase the difficulty and risk of removal of the correction plate; therefore, how to properly fix the bar is also an important issue [15, 17].

Various modified methods and their related risks have been described for reducing the complications involving the patient’s history, specially designed and customized instruments, or modifications to the procedure (see Table 3) [11, 17–25]. The modifications in the surgical procedure could be classified into four major categories: (1) patient’s position during surgery, (2) wound incisions, (3) bending of the end of the bar before removal, and (4) bar rotation during removal [24]. The positions are classified as supine, prone [22], lateral [21], and special two-table supine positions [20, 23]. All our patients were in the supine position during the operation. We suggest adopting the supine position in a single bed (Fig. 1) which is relatively easy to prepare and can be used to smoothly cooperate with subsequent surgical procedures. Incisions are divided into unilateral (right or left side) or bilateral incision. We suggest that a bilateral incision be made on both sides of the previous Nuss procedure to better observe whether a callus has formed or if the steel wire is broken on both sides of the steel plate. If procedures are present with more than one bar insertion or bar fixed on both ends, a bilateral approach is necessary. The steel plate is suggested to be removed on the same side from which the steel plate was placed previously. The approach for bending the end of the pectus bar is divided into unilateral, bilateral, or no bending. We suggest using unilateral partial bending, i.e. bending one side of the steel plate first and then removing the steel plate from the opposite side, which can reduce the risk of injury to viscera in the thoracic cavity and pleural cavity caused by the bent steel plate. Regarding the device, the pectus removal bender from the original manufacturer would suffice. Rotation is divided into partial or no rotation. We suggest no rotation during removal to prevent unnecessary injuries or bleeding. Besides, we suggest wrapping an elastic bandage around the chest for 3 days after the surgery to reduce the dead space and prevent wound hematoma formation (Fig. 2). In addition, most of our patients were adults, and the majority of patients had more than one pectus bar placed. These were considered the risk factors associated with higher complications. In our results, the adults and number of bars removal were not risk factors.

**Table 3:** Review of major articles reporting the bar removal after Nuss procedure

Study (year)	Number of patients (number of bar)	Age (years), mean ± SD (range)	Interval (years) mean ± SD (range)	Operation technique	Complications
Bilgi et al. (2017) [11]	246 (1 bar: 162; 2 bars: 80; 3 bars: 4)	17.7 ± 6.2 (age of repair)	2.88 ± 1.43	Position: supine Incision: bilateral Straightening: bilateral Others: Subcutaneous drain for preventing seroma by surgeon’s discretion.	Seroma: 29 (11.7%); pneumothorax: 3 (1.2%); pleural effusion: 2 (0.8%); secondary intervention: 6 (2.4%; 3 massive bleeding). Risk factor: double bars removal
Park et al. (2016) [22]	1821 (NM*)	9.13 (1.3–44, age of repair)	2.57 (0.3–14). 2.02 for < 12 years; 2.99 for 12–20 years; 3.53 for > 20 years	Position: supine Incision: bilateral Straightening: bilateral Others: osteotome, rongeur dissection or electric drilling for removal callus. Sternal wire for malpositioned pectus bars, or crane elevation of the sternum.	Seroma/infection: 43 (2.36%); pleural effusion: 3 (0.16%); bleeding: 3 (0.16%; 1 cardiopulmonary bypass for hemostasis); hemothorax: 1 (0.05%)
Liu et al. (2013) [18]	186 1 bar: 184 2 bars: 2	9.8 (5–26) (age of removal)	2 years: 133 ≥2.5 years: 53	Position: supine Incision: right side Straightening: no Others: the tip of the bar grafted with a bar flipper, and the flipper was turned several times in the clockwise and counterclockwise direction to loosen the bar from the surrounding fibrous capsule	Pneumothorax: 3 (1.6%)
Nyboe et al. (2011) [21]	334 1 bar: 281 2 bars: 53	19.1 (age of bar removal)	3.12 (1.76–7.05).	Position: supine Incision: unilateral (*n* = 218); bilateral (*n* = 116) Straightening: not routine Other: postoperative X-ray not as a routine	Pneumothorax: 5 (1.4%;); hemothorax: 3 (1.0%; 1 requiring open surgery, 2 treated with a chest tube)
Fike et al. (2012)	230 (NM)	16.7 (7.8–25.3) (age of bar removal)	2.8 (0.9–9.2	Position: supine; two tables with T-shape Incision: bilateral Straightening: no	Wound infection: 6 (3%); Massive bleeding: 1 (0.4%; with blood transfusion)
Chon et al. (2011) [20]	21 (NM)	NM	NM	Position: prone Incision: unilateral Straightening: no	No complication
Varela et al. (2010) [24]	21 (NM)	NM	NM	Position: lLateral (20); supine (1) Incision: Unilateral (20); bilateral (1) Straightening: No (20); yes (1; unilateral)	No complication
de Campos et al. (2009) [17]	14 (NM)	NM	NM	Position: supine Incision: bilateral Straightening: bilateral Others: using a protective film around one end of bar	Intraoperative bleeding: 1 (surgical exploration)
St Peter et al. (2007) [19]	110 (NM)	NM	NM	Position: supine; two tables with T-shape Incision: bilateral Straightening: no	No complication
Fujita et al. (2005) [25]	10 (1 bar: 10)	NM	NM	Position: supine Incision: bilateral Straightening: bilateral	No complication

*NM* not mentioned

In our experience, no major complications occurred after the procedure. There was no statistically significant correlation between the age groups and the number of pectus bar removed. Postoperative pneumothorax was found in six patients. One patient needed intraoperative pleural drainage due to one end of the bar being trapped to the lungs. Others had no clinical symptoms or an increase in the hospital stay. Pneumothorax mostly occurred due to air entering the pleural space via the wound during dissection for the bar removal. It can be resolved progressively without drainage if there are no other clinical symptoms. Otherwise, if the pneumothorax is due to the lung injury, pleural drainage should be done.

The mean intraoperative blood loss was less than 12 mL. Ten patients had perioperative blood loss of 50–100 mL. The bleeding was from the callus or newly grown vessels and was controlled carefully. No patient needed blood transfusion. We found that callus formation causes significantly more perioperative bleeding and longer operative time (Table 2). We suggest that with severe callus formation around the ends of bar, careful dissection and bleeding control be undertaken. Furthermore, to prevent traction of the mediastinal adhesions when pulling out the bar blindly, the callus covering the ends of bar should be completely removed and the bar should be removed along with the shape of the thoracic cage without rotation. Life-threatening complications such as organ injuries and major bleeding could be avoided.

The insufficient number of patients is the main limitation of this study. Other limitations include the risk of bias inherent to the retrospective design of the study and the inability to generalize the results and conclusions to other populations. Bleeding volume during surgery was also not recorded carefully. Except for a large amount of bleeding, the estimated bleeding volumes were recorded as < 10 mL. To facilitate statistics, these were calculated as 10 mL. Therefore, the average bleeding volume in this study is higher than that in other studies.

## Conclusion

Adult patients and removal of multiple bars were not the risk factors for the occurrence of complications in our cohort. Skills in preventing intraoperative mediastinal traction, carefully controlling bleeding, and reducing the effect of dead space around wounds could minimize the risk of bleeding complications. A multicentric study or the accumulation of more cases is needed to further evaluate the risk factors of removal pectus bar(s).

## Data Availability

• All data generated or analysed during this study are included in this published article. • The datasets generated and/or analysed during the current study are not publicly available due the consideration of patient data confidentiality and subsequent research but are available from the corresponding author on reasonable request.

## Abbreviations

BMI: Body mass index
CWIG: Chest Wall International Group
MIRPE: Minimally invasive repair of pectus excavatum
PE: Pectus excavatum
